# Editorial: Progressive role of artificial intelligence in treatment decision-making in the field of medical oncology

**DOI:** 10.3389/fmed.2025.1701096

**Published:** 2025-10-09

**Authors:** Nishanth Thalambedu, Barath Prashanth Sivasubramanian, Sumit Gupta, Mamtha Balla

**Affiliations:** ^1^University of Arkansas for Medical Sciences, Little Rock, AR, United States; ^2^Northeast Georgia Health System Inc, Gainesville, FL, United States; ^3^Cure 4 The Kids Foundation, Las Vegas, NV, United States; ^4^Department of Hematology and Oncology, The University of Toledo, Toledo, OH, United States

**Keywords:** artificial intelligence (AI), oncology, biomarker, lung cancer (LC), breast cancer, colon cancer

Artificial intelligence (AI) is rapidly transforming medical oncology. Since the FDA approval of several AI-driven tools, their integration into clinical practice has shown clear benefits in improving diagnostic accuracy, optimizing treatment decisions, and enhancing patient outcomes (1). Unlike conventional models, AI has the ability to rapidly process and learn from high-dimensional data, offering unparalleled opportunities for personalization of cancer therapy and predictive modeling (2). This Research Topic presents nine seminal contributions that collectively elucidate the role of AI and novel biomarkers in improving cancer detection, diagnosis, treatment personalization, and disease monitoring across diverse malignancies.

Thalambedu et al. provided a comprehensive review of circulating tumor DNA (ctDNA) in non-small cell lung cancer (NSCLC). The authors provided a detailed summary of the biological underpinnings, isolation techniques, and clinical applications of ctDNA. Notably, baseline ctDNA levels were shown to correlate significantly with tumor burden and prognostic outcomes such as progression-free survival (PFS) and overall survival (OS). The review highlights recent advances in sequencing technology combined with AI analytics enabling detection of mutant allele fractions as low as 0.05%, facilitating early diagnosis and minimal residual disease (MRD) detection with sensitivities reported as high as 90% and specificities approximating 95%.

Expanding on biomarker integration, Bongurala et al. reviews the application of multi-omics data—including ctDNA, exosomal RNA, and proteomic signatures—in conjunction with AI bioinformatics. This synthesis reportedly enhances early cancer detection sensitivity by over 30% relative to single-analyte assays, underscoring the potential of multidimensional biomarker approaches for improved diagnostic precision.

The clinical utility of AI in pediatric oncology is exemplified in by Lozano et al. which focused on neuroblastoma by utilizing the PRIMAGE database and developed a machine learning model integrating clinical, molecular, and magnetic resonance (MR) radiomics features for overall survival prediction and risk stratification. The random survival forest model demonstrated robust performance with a concordance index of 78.8 ± 4.9 and a time-dependent area under the curve (AUC) of 77.7 ± 6.1 in the test set, and even higher metrics in the independent cohort (C-index 93.4, AUC 95.4). Importantly, lesion heterogeneity, size, and molecular variables were identified as key prognostic factors. This AI-driven model provided superior stratification into low-, intermediate-, and high-risk groups compared to the current standard-of-care classification, enabling more precise categorization of patients, thereby offering significant potential for guiding personalized therapeutic approaches (Lozano-Montoya et al.). AI systems are also helping in target identification and finding possible candidates for new targeted therapy in pediatric tumors. Analysis of circulating tumor DNA and methylation data are helping in developing efforts for early detection and liquid biopsies (3). Establishing diagnosis using the images from pathology slides in leukemia to classification of brain tumors based on radiological features, AI is playing an important role in pediatric tumors (4).

Therapeutic optimization is further demonstrated by Sigawi et al. where an AI-driven personalized dosing regimen for lenvatinib in thyroid and salivary gland cancers, using clinical and pharmacokinetic data to optimize dosage. The approach reduced dose-related toxicities by 25% without compromising treatment efficacy, demonstrating the clinical value of computational decision-support tools in improving therapeutic tolerability and patient adherence (Sigawi et al.).

Surgical management considerations are addressed by Zhang et al. by reporting AI-based predictive models integrating clinical and molecular parameters to inform primary tumor resection decisions in metastatic colorectal cancer. The model achieved an area under the receiver operating characteristic curve (AUC) of 0.87, effectively stratifying patients for surgical benefit, with estimated survival improvements of 15–20% in selected cohorts.

In the context of early cancer detection, Chandramohan et al. reviewed AI-assisted imaging modalities for breast cancer screening. The authors report enhancements in mammographic sensitivity by 12%, alongside a 20% reduction in false positive rates, thereby improving diagnostic accuracy and reducing unnecessary interventions (Chandramohan et al.).

Extending the discussion of imaging and early detection, Bhattacharya et al. explore the role of regenerative AI in breast cancer care across diagnosis, treatment, drug discovery, and monitoring. Tools like LYNA and Mia improve imaging sensitivity up to 99%, while platforms such as Tempus and IBM Watson enable tailored treatments by integrating clinical and genomic data. In drug development, AI models from Insilico and Atomwise accelerate therapeutic discovery. Remote monitoring platforms like MyPathway and Flatiron support real-time care adjustments, and innovations like MammoAssist and Niramai expand early detection in low-resource settings. Despite challenges like data quality and transparency, the article emphasizes AI's broad potential to improve outcomes and access globally.

Predictive modeling in endocrine oncology is exemplified by Firat et al. which introduces a hybrid relational classification model for forecasting recurrence in differentiated thyroid cancer (DTC) by incorporating 13 clinicopathological features, the study employed SMOTE-NC for class balancing and developed two models—RCAR and CBAR. The RCAR model achieved superior accuracy (96.7%), sensitivity (93.1%), and F1 score (96.7%). Association rules identified that papillary histology with incomplete treatment response and lymphadenopathy were strong predictors of recurrence. In contrast, complete response and absence of adenopathy predicted durable remission. This robust, interpretable model offers actionable insights for individualized follow-up strategies in DTC care.

Finally, Zhu et al. provide a comprehensive overview of AI applications across the colorectal cancer (CRC) continuum, from early detection to prognosis. AI-enhanced colonoscopy tools like CADe and CNNs improve polyp detection up to 94%, while EndoBRAIN matches expert diagnostic accuracy. In pathology and staging, CNNs and MRI radiomics models predict lymph node metastases and extramural venous invasion with high AUCs (up to 0.925). AI-driven robotic surgery and MRI-based radiotherapy models further optimize treatment response. Prognostic tools leveraging tumor-stroma ratios offer added survival prediction value. These advances collectively improve precision and personalization in CRC care.

Collectively, the articles in this Research Topic advance the understanding of how AI and biomarker innovations can synergistically enhance oncologic care. They demonstrate measurable improvements in early detection, precise diagnosis, personalized therapy, and dynamic disease monitoring, while acknowledging ongoing challenges related to data standardization, model interpretability, cost-effectiveness, and equitable access.

The integration of AI with biomarker-driven approaches represents a pivotal axis of progress in precision oncology. Multidisciplinary collaboration and rigorous clinical validation will be paramount to fully realize the potential of these technologies in improving patient outcomes globally. This compilation of scholarly work provides a timely and insightful synthesis that charts the trajectory toward a more intelligent, effective, and accessible cancer care paradigm.
